# Multifunctional self-assembled composite colloids and their application to SERS detection†
†Electronic supplementary information (ESI) available: Experimental part; magnetic separation and Raman scattering characterization of micelles; additional TEM images; and analysis of the effect of surface charge on SERS detection. See DOI: 10.1039/c5nr01264c



**DOI:** 10.1039/c5nr01264c

**Published:** 2015-05-06

**Authors:** Andrea La Porta, Ana Sánchez-Iglesias, Thomas Altantzis, Sara Bals, Marek Grzelczak, Luis M. Liz-Marzán

**Affiliations:** a Bionanoplasmonics Laboratory , CIC biomaGUNE , Paseo de Miramón 182 , 20009 Donostia – San Sebastián , Spain . Email: llizmarzan@cicbiomagune.es; b EMAT-University of Antwerp , Groenenborgerlaan 171 , B-2020 Antwerp , Belgium; c Ikerbasque , Basque Foundation for Science , 48013 Bilbao , Spain; d Department of Chemistry , College of Science , King Saud University , 11451 Riyadh , Kingdom of Saudi Arabia

## Abstract

We present a simple method for the co-encapsulation of gold nanostars and iron-oxide nanoparticles into hybrid colloidal composites that are highly responsive to both light and external magnetic fields. Self-assembly was driven by hydrophobic interactions between polystyrene capped gold nanostars and iron oxide nanocrystals stabilized with oleic acid, upon addition of water. A block copolymer was then used to encapsulate the resulting spherical colloidal particle clusters, which thereby became hydrophilic. Electron microscopy analysis unequivocally shows that each composite particle comprises a single Au nanostar surrounded by a few hundreds of iron oxide nanocrystals. We demonstrate that this hybrid colloidal system can be used as an efficient substrate for surface enhanced Raman scattering, using common dyes as model molecular probes. The co-encapsulation of iron oxide nanoparticles renders the system magnetically responsive, so that application of an external magnetic field leads to particle accumulation and limits of detection are in the nM range.

## Abstract 2

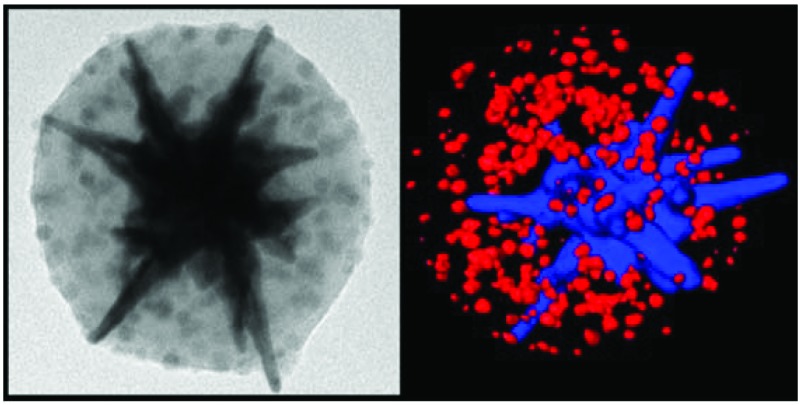
Development of a novel hybrid colloidal system suitable for surface-enhanced Raman spectroscopy analysis.

## Introduction

The combination of several functions within a single colloidal object has been proposed as a means to expand the potential applications of nanomaterials. A prototypical example of this class of systems is the integration of optical and magnetic properties, which has been achieved *via* different strategies.^1–6^ Most of these methods however require either chemical reactions to be carried out on pre-formed colloids, or the irreversible assembly/aggregation of particles that are synthesized independently. The former method usually leads to poorly defined morphologies, whereas the latter results in rather large supra-particles. Recent progress on directed self-assembly has demonstrated that application of hydrophobic interactions may lead to the reversible assembly of plasmonic nanoparticles of various morphologies and sizes.^7^ An additional challenge was the application of the same concept to the reversible assembly of particles with dissimilar composition and surface chemistry. We were interested in applying this concept toward the formation of multifunctional assemblies that could retain small sizes, which are required for example in most biological applications. In particular, the incorporation of plasmonic and magnetic nanoparticles offers several advantages, such as multimodal imaging capabilities or magnetic manipulation and increased detection sensitivity. A similar concept has been applied in the past toward the improvement of sensing and detection based on surface enhanced Raman scattering (SERS).^8,9^ For example, microgels containing iron oxide and silver nanoparticles were shown to allow the capture of various analytes and ultrasensitive detection,^10^ whereas silica-coated iron oxide spindles covered with a dense layer of gold nanorods could be accumulated on a tiny spot, increasing SERS sensitivity as compared to gold nanorods alone.^11^ The latter case is based on the aggregation of the nanoparticles induced by an external magnetic field, so that additional hot-spots are created and a larger amount of sample is probed, leading to a lower limit of detection when using very small amounts of analytes. The recent development of colloidal synthesis methods has allowed a detailed comparison of the efficiencies of metal nanoparticles with different morphologies as SERS enhancers. It is commonly accepted that spheres are only efficient when forming aggregates,^12–14^ whereas nanorods,^15–17^ nanocages or nanoplates^18–20^ can perform even as single particles in solution. The most efficient performance thus seems to be related to the presence of sharp tips and therefore nanostars have been identified as displaying the optimum morphology for SERS enhancement.^21–23^

In this context, we present here a method to prepare colloidal hybrid assemblies that are stable in aqueous solution, while displaying interesting optical and magnetic properties, by co-encapsulation of gold nanostars and iron oxide nanocrystals within a protecting layer of poly(styrene)-*block*-poly(acrylic acid) (PS-*b*-PAA). Such a hybrid assembly combines the plasmonic properties characteristic of AuNSs with the response to external magnetic fields provided by the presence of the superparamagnetic iron oxide NPs. This magnetic–plasmonic system was applied to the SERS detection of model molecular dyes. Interestingly, by applying magnetic accumulation, limits of detection in the nM range could be achieved.

## Results and discussion

**Fig. 1:**
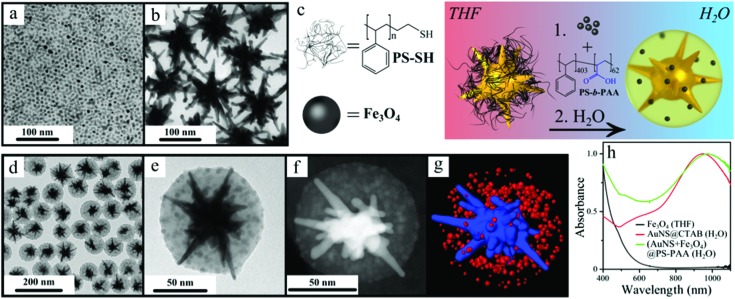
(a,b) TEM images of oleic acid-capped iron oxide NPs (a) and polystyrene-stabilized AuNSs (b). (c) Scheme illustrating the encapsulation process. (d,e) TEM images of hybrid colloidal assemblies at different magnifications. (f) HAADF-STEM image of an individual composite particle. (g) Three-dimensional electron tomography reconstruction of an individual cluster showing the gold nanostar in blue and iron oxide nanoparticles in red. (h) UV-Vis spectra of CTAB-stabilized gold nanostars in water (red line), iron oxide nanoparticles stabilized with oleic acid in THF (black line) and the composite clusters in water (green line).

The starting building blocks for the targeted composite colloids are gold nanostars (AuNSs)^24^ and iron oxide nanoparticles (Fe_3_O_4_NPs).^25^ Fe_3_O_4_NPs (7.0 ± 0.6 nm, Fig. 1a) were stabilized with oleic acid and thus were intrinsically hydrophobic after synthesis and stable in pure THF. AuNSs (110.2 ± 16.1 nm, Fig. 1b) were initially capped with the cationic surfactant cetyltrimethylammonium bromide (CTAB) and subsequently mixed with an excess (∼5 molecules per nm^2^) of thiol-terminated polystyrene (PS, *M*_w_ = 53 kg mol^–1^), leading to ligand exchange. This process yielded hydrophobic AuNSs that were also colloidally stable in THF.

The formation of magnetoplasmonic assemblies was based on a recently reported encapsulation strategy, in which polymeric micelles play a role of protecting layers.^26^ The process involves the addition of water (10 wt%) to the THF solution containing a stable mixture of gold nanostars, iron oxide nanoparticles and the linear block copolymer (PS-*b*-PAA) (Fig. 1c). Water addition resulted in a gradual variation of the polarity of the mixture, thereby driving co-aggregation of all three components mediated by hydrophobic interactions. To ensure the incorporation of Fe_3_O_4_NPs at the void space between the spikes of AuNSs, an excess of magnetic nanoparticles was used as compared to nanostars. Since hydrophobic interactions are not directional, the resulting assembled mixture contained three different types of colloidal nanostructures after aggregation: (i) empty copolymer micelles, (ii) polymer-encapsulated spherical aggregates of Fe_3_O_4_NPs, and (iii) single AuNSs co-encapsulated within polymeric micelles containing a large number of small Fe_3_O_4_NPs. Because of the larger mass of AuNSs, centrifugation could be readily used to remove both empty micelles and micelles containing iron oxide nanoparticles only, effectively resulting in the purification of the bifunctional clusters (Fig. 1d). The final product thus consisted of individual gold nanostars co-encapsulated with surrounding magnetic nanoparticles. These encapsulated magnetic–plasmonic micelles exhibit excellent colloidal stability in water because the outer polymer layer comprises the hydrophilic PAA blocks.^27^

Morphological characterization by transmission electron microscopy (TEM) confirmed that each nanostar was individually encapsulated in a polymeric micelle (143.1 ± 11.2 nm total diameter), which also contained clearly visible spots corresponding to Fe_3_O_4_NPs (Fig. 1e). In addition, high angle annular dark field scanning TEM (HAADF-STEM) was used for detailed analysis of individual clusters. As shown in Fig. 1f, the micelle comprises a bright central object corresponding to gold nanostars, surrounded by satellite iron oxide nanoparticles. Electron tomography and three-dimensional reconstruction of the same particle provided clear information on the spatial distribution of the nanoparticles in three dimensions. As shown in Fig. 1g, the AuNS (in blue) and the Fe_3_O_4_NPs (in red) are homogeneously distributed within the inner space of the micelles. Interestingly, the spikes of the AuNSs consistently appear to stick out of the polymeric envelope, as previously reported for single AuNSs.^26^ The gaps between the particles confirm the presence of polymeric capping agents, while image analysis in 3D allowed us to estimate the presence of 200–300 Fe_3_O_4_NPs per cluster.

The metallic cores of the magnetoplasmonic assemblies define the optical response of the system. As compared with individual AuNSs, increased absorbance was registered below 600 nm, due to enhanced light scattering in the presence of the polymeric shell, as well as some absorption by Fe_3_O_4_NPs, at wavelengths shorter than the localized surface plasmon resonances (LSPR) of gold nanostars (Fig. 1h). On the other hand, the copolymer shell and the oxide nanoparticles increase the refractive index around the surface of the nanostars, leading to a redshift of the LSPR maximum by 55 nm.^26^ The magnetic response of the magnetoplasmonic micelles can be readily observed by the naked eye. Application of a hand-held magnet for 2 hours next to the colloid containing the hybrid clusters induces a clear separation of the particles and accumulation on the vial wall (Fig. S1†).

**Fig. 2:**
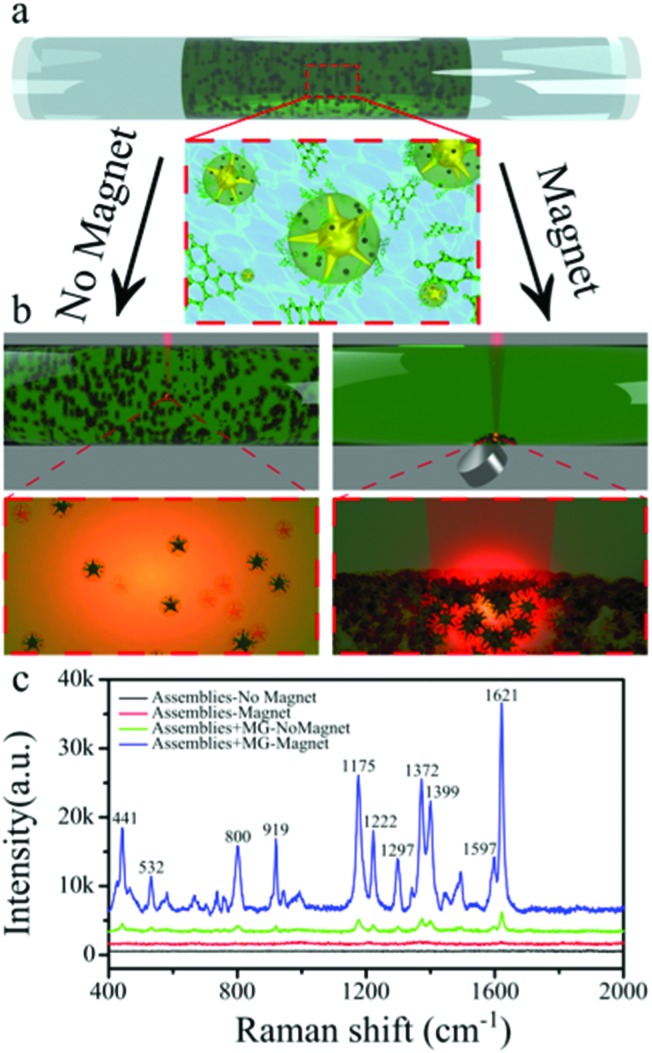
Schematic representation of the experimental setup for magnetic field assisted SERS detection. The mixture of hybrid clusters and analyte is placed inside a glass tube (a), SERS measurements are performed either in the presence or in the absence of a handheld magnet (b). SERS spectra in (c) show a strong signal enhancement upon application of the external magnetic field and particle accumulation. The flat spectra of the assemblies without analyte (black and red lines) confirm that the recorded peaks stem from Malachite Green (MG).

The sharp spikes of AuNSs branching out of the polymeric micelle were expected to provide sufficient electric field enhancement to serve as efficient substrates for SERS. This was analysed by means of the experimental design schematically shown in Fig. 2. In a typical experiment, the mixture (20 μL) containing hybrid colloidal clusters and the desired concentration of analyte molecules was placed inside a glass tube (∼1 mm internal diameter) and sealed at both ends (Fig. 2a). Such an experimental configuration was designed to prevent solvent evaporation and ensure a constant concentration of the analyte during the entire measurements. SERS spectra were acquired in the absence and in the presence of an external handheld magnet. It is worth mentioning that the laser focal point was adjusted in each case, so that in the absence of the magnetic field the focus was at the center of the tube but upon application of the magnet it was shifted to the bottom of the tube, where particles aggregate (Fig. 2b). The small volume of the sample and the strong magnetic response of the particles allowed complete aggregation of the particles and accumulation at the glass tube wall within a short time, ∼2 minutes.

Representative SERS spectra are displayed in Fig. 2c before and after addition of Malachite Green (MG), with and without an applied magnetic field, revealing a significant intensity difference depending on the aggregation state of the nanoparticles. Under an external magnetic field, the hybrid clusters form a compact aggregate at the inner wall, leading to signal accumulation and an increase of the electric near field, thereby further enhancing the SERS signal.^28,29^ To avoid potential contamination of the SERS signal by polystyrene peaks the laser power was kept relatively low (0.61 mW) and an integration time of 10 s was used. Under such conditions no peaks from polystyrene were observed even after magnetic field accumulation (for more detailed analysis of polystyrene SERS peaks, see Fig. S2†). This is clearly exemplified for MG in Fig. 2, as its primary vibrations can be readily identified in the SERS spectra, related to stretching (C–C) at 1297, 1597 and 1621 cm^–1^, stretching (*N*-phenyl) at 1372 and 1399 cm^–1^, in plane bending (C–H) at 1175 and 1222 cm^–1^, ring skeletal vibration of radical orientation at 532 and 919 cm^–1^, out of plane bending (C–H) at 800 cm^–1^ and out of plane bending (phenyl–C^+^–phenyl) at 441 cm^–1^.^30,31^ For a more detailed peak assignment see Table S1.† Although the peaks for MG are present both with and without an applied magnetic field, a significant signal enhancement was obtained by magnetic accumulation. These results thus confirm that the applied magnetic field can remotely activate the formation of hot spots and increase the analyte concentration, thus providing a convenient scenario for ultrasensitive detection.

**Fig. 3:**
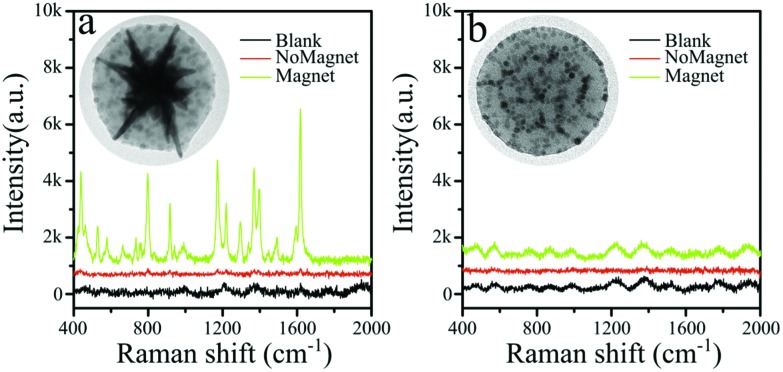
SERS spectra of MG in the presence of magnetic-plasmonic micelles (a) and magnetic micelles (b). The presence of the plasmonic AuNS core is crucial to sufficiently enhance the Raman signal. The blanks correspond to a magnetically aggregated sample with no dye.

The contribution toward enhancement of the Raman signal by the local increase of analyte molecules and by hot spot formation in between gold nanostars was further studied using magnetic micelles containing iron oxide nanoparticles only (without gold nanostars; see detailed synthesis information and TEM image in the ESI†). These AuNS-free micelles respond to the external magnetic field in a similar fashion to those containing AuNS. Fig. 3 shows a comparison of Raman and SERS spectra for both micellar systems. In the absence of a magnetic field no Raman peaks were recorded for the solution containing 10 nM MG. Magnetic accumulation of the assemblies makes possible the detection of analyte molecules only when the micelles containing AuNSs together with Fe_3_O_4_ NPs were used (Fig. 3a and b). These results confirm the role of gold nanostars as essential components of the SERS substrates. By combining magnetic and optical activity, the analyte concentration at the illumination volume can be increased by magnetic accumulation and exploit the surface plasmon electric field enhancement at the AuNS tips for efficient SERS.

Since the outer PAA polymer shell displays a negative value of *ζ*-potential (–25 mV), we hypothesize that analyte molecules with negative functional groups will be repelled from the micelle surface whereas molecules with positive functional groups absorb onto the outer polymer surface. Such different interactions between analyte molecules and the polymer surface ultimately determine the ability of the clusters for magnetic separation and SERS detection. This hypothesis was tested by comparing Malachite Green as a positively charged probe and Trypan Blue (TB) as a negatively charged analyte. A mixture containing the colloidal clusters and the analyte molecules (10^–5^ M) was decanted with the help of an external magnet and then redispersed in pure water. The washing cycle was repeated three times. After each washing step, SERS spectra were recorded in the presence and the absence of an external magnetic field. We found for MG that the peak intensity was higher under a magnetic field, indicating co-accumulation of the analyte molecules and magnetic particles. After subsequent washing steps, the peak intensity progressively decreased (Fig. S4†). The peak intensity however remained constant (nearly zero) after washing when no magnetic field was applied. The difference of Raman intensities between aggregated and dispersed modes suggests that MG molecules do adsorb onto the surface of the micelles, but can be gradually washed away. TEM analysis additionally confirmed the formation of a molecular shell around the hybrid clusters after treatment with MG molecules (Fig. S5†). In the case of negatively charged TB, we found that the SERS intensities from the initial mixture were higher in the absence of a magnetic field (Fig. S4†). This counter-intuitive observation is explained by the fact that the magnetic aggregation of the clusters may lead to expelling of weakly bound TB molecules to the bulk solution. This behaviour thus suggests that the micelles cannot accumulate TB molecules by induced phase separation. After the first washing step, the Raman intensity dropped to zero regardless of the external magnetic field, confirming the lack of retention of the analyte molecules on the surface of magnetoplasmonic micelles. These results constitute a step toward understanding the surface chemistry of the clusters and may be of help when devising biomolecule detection.

**Fig. 4:**
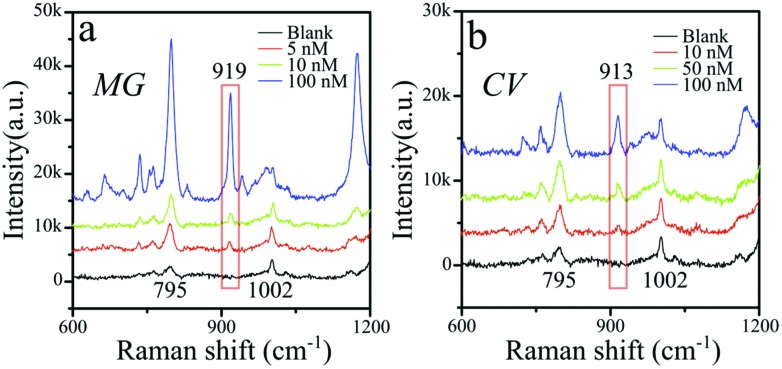
Limit of detection analysis under magnetic field accumulation and higher laser intensity (5.93 mW). Limits of detection are found to be 5 nM for MG (a) and 10 nM for CV (b).

We finally evaluated the detection limit for MG and crystal violet (CV) as model (positively charged) probe molecules (Fig. 4). The SERS spectra were recorded under an applied magnetic field taking as a reference the intensity of the 919 cm^–1^ and 913 cm^–1^ peaks for MG and CV, respectively. These peaks were selected to avoid potential interference with the peaks from polystyrene (795 cm^–1^, 1002 cm^–1^ and 1200 cm^–1^), which are visible at the relatively high laser power (5.93 mW) and prolonged accumulation time (30 s) used in these experiments. The peaks corresponding to polystyrene however could only be detected in the presence of an external magnetic field. Under these conditions we obtained safe limits of detection of 5 nM for MG and 10 nM for CV, thus confirming the ultradetection capability of this system.

## Conclusions

We have demonstrated that the concept of hydrophobic interactions as a driving force toward the reversible assembly of nanoparticles can be safely applied to the co-encapsulation of nanoparticles with dissimilar size, morphology, composition and surface chemistry. Simple addition of water to a mixture of gold nanostars and iron oxide nanoparticles in THF allowed us to develop novel multifunctional nano-platforms that are responsive toward optical and magnetic stimuli, which was applied to SERS-based ultradetection. While the incorporation of a large amount of magnetic nanoparticles into each capsule enables remote accumulation of the adsorbed analyte molecules, the plasmonic gold nanostars act as highly efficient substrates to enhance the Raman scattering signal, achieving detection limits in the nM regime.
