# Supplementary material for: Multifunctional self-assembled composite colloids and their application to SERS detection
Source: Nanoscale. 2015 May 6;7(23):10377–81. doi: 10.1039/c5nr01264c (PMC4774408; doi:10.1039/c5nr01264c)
Supplement: Supplementary file 1 [file NR-007-C5NR01264C-s001.pdf]

Electronic Supplementary Material (ESI) for Nanoscale.
This journal is © The Royal Society of Chemistry 2015

Multifunctional Self-Assembled Composite Colloids and their Application to SERS Detection

Electronic Supplementary Information

Andrea La Porta,[a] Ana Sánchez-Iglesias,[a] Thomas Altantzis,[c] Sara Bals,[c] Marek Grzelczak,[a,b] and Luis M. Liz-Marzán[a,b,d,]*

[a]Bionanoplasmonics Laboratory, CIC biomaGUNE, Paseo de Miramón 182, 20009 Donostia – San Sebastián, Spain.
[b]Ikerbasque, Basque Foundation for Science, 48013 Bilbao, Spain.
[c]EMAT-University of Antwerp, Groenenborgerlaan 171, B-2020 Antwerp, Belgium
[d]Department of Chemistry, College of Science, King Saud University, 11451 Riyadh, Kingdom of Saudi Arabia.

S1. Experimental Part 1
S2. Magnetic properties of hybrid clusters 3
S3. Raman characterization of hybrid clusters without analyte 3
S4 Magnetic clusters 4
S5. Effect of analytes charge on SERS performance 5
References 6

S1. Experimental Part

Chemicals. Hydrogen tetrachloroaurate trihydrate ($HAuCl_4 \cdot 3H_2O$, ≥99.9%), silver nitrate ($AgNO_3$, ≥99.0%), L-ascorbic acid (≥99%), iron chloride ($FeCl_3 \cdot 6H_2O$ ≥99.9%), hydrochloric acid (HCl >99%) and sodium oleate were purchased from Aldrich. Thiol-terminated polystyrene (PS-SH) with a molecular weight of 53 kg/mol and diblock copolymer (polystyrene-b-polyacrylic acid, PS_{403}-b- PAA_{62}) were purchased from Polymer Source, Inc. All chemicals and solvents were used without further purification.

Synthesis of gold nanostars. The surfactant-free method was used to prepare Au nanostars.[1] A solution of gold seeds (~15 nm, [Au] = 0.5 mM, 0.5 mL) prepared by the Turkevich method[2] was added to a solution (50 mL) containing $HAuCl_4$ (0.25 mM) and HCl (1 mM), followed by addition of $AgNO_3$ (10 mM, 0.15 mL) and ascorbic acid (100 mM, 0.25 mL). To increase the stability of the obtained nanostars, CTAB (100 mM, 2.5 mM) was added to the growth solution. Upon synthesis, the solution was centrifuged (4500 rpm, 30 min) to remove excess reactants, and redispersed in water.

Ligand exchange. The corresponding gold nanostar colloid (5 mM, 1 mL) was added dropwise under sonication to a THF solution (10 mL) of PS-SH (5 molecules of PS-SH per nm^2 of gold surface). The solution was kept in an ultrasonic bath for 15 min. To ensure ligand exchange, the resulting mixture was left undisturbed for 12h, and then centrifuged twice. The particles were finally dispersed in THF (final concentration 5 mM).

Synthesis of iron oxide nanoparticles. Iron oxide nanoparticles were prepared according to the method reported by Park et al.[3] Final concentration of Fe_3O_4 NPs in THF was 62 mg/mL.

Self-assembly of hybrid micellar clusters

In a typical assembly experiment, water (1 mL) was added dropwise to a mixture in THF containing AuNSs@PS (1.9 mL, 5.5 mM), PS_{403}-b- PAA_{62} (0.2 mL, 1.5 mg/mL) and Fe_3O_4 NPs (0.01 mL, 62 mg/mL) under magnetic stirring. Subsequently, the water content was increased up to 50 wt%, followed by increasing the temperature up to 50 ºC, which was maintained for 30 min. The final solution was centrifuged twice (3500 rpm, 20 min) and the particles redispersed in pure water at a concentration of [Au] = 0.5 mM.

Self-assembly of magnetic micelles

Water (1 mL) was added to a mixture in THF containing PS_{403}-b- PAA_{62} (0.2 mL, 1.5 mg/mL) and Fe_3O_4NPs (0.01 mL, 62 mg/mL) under magnetic stirring. Subsequently, the water content was increased up to 50 wt%, followed by increasing the temperature up to 50 ºC, which was maintained for 30 min. The final solution was centrifuged twice (3500 rpm, 20 min) and the particles dispersed in pure water.

Sample preparation and SERS measurements

The hybrid colloid ([Au] = 0.5 mM) and the desired amount of analyte were incubated for 2 hours to reach thermodynamic equilibrium. A small amount (20 µL) of the sample was then used to fill a thin glass tube (~1 mm internal diameter), which was sealed at both ends by parafilm®. A magnetic field we applied by putting a small commercial handheld magnet placed in contact with the tube. In order to avoid fluctuations in the SERS intensity due to the flow of NPs following the magnetic field, each sample was left in contact with the magnet during 15 minutes prior to recording the spectra. After this time no relevant changes in the intensities were observed.

Characterization

Optical extinction spectra were recorded using an Agilent 8453 UV/Vis diode-array spectrophotometer. SERS spectra were recorded using a Renishaw InVia Raman microscope equipped with two Peltier-cooled CCD detectors, a Leica microscope with two gratings of 1200 and 1800 lines/mm and band-pass filter optics. Excitation lasers with emission wavelengths of 633 and 532 nm were used and focused onto the sample through a 10× objective with N.A. 0.25, producing spot diameters of 3.1 and 2.6 µm for 633 and 532 nm, respectively. Except for the limit of detection experiment, all SERS spectra were collected with an integration time of 10 s, and the samples were irradiated with constant powers of 0.61 mW (633 nm) and 2.43 mW (532 nm). For the detection limit study, the samples were irradiated with 633 nm excitation at a constant power of 5.93 mW and 30s integration time. Transmission electron microscopy (TEM) images were collected with a JEOL JEM-1400PLUS instrument operating at 120 kV. HAADF-STEM images and electron tomography tilt series were acquired using a double aberration corrected cubed FEI Titan 50-80 electron microscope operated at 300kV. For the reconstruction of the series we used the SIRT algorithm, as implemented in the ASTRA toolbox.[4,5]

S2. Magnetic properties of hybrid clusters

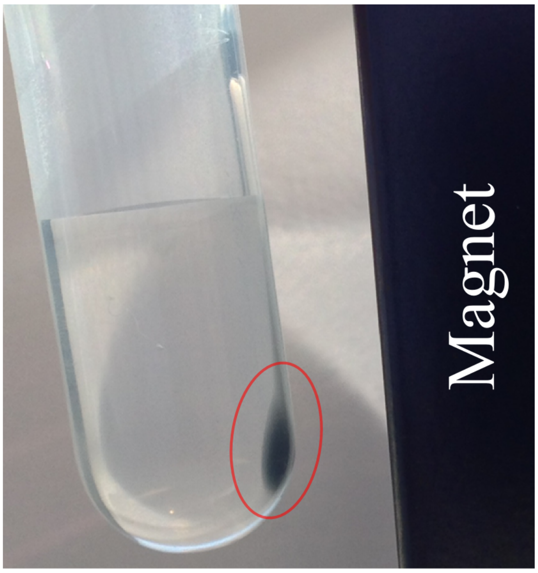

Figure S1. The stable colloid of hybrid clusters phase separates upon application of an external magnetic field.

S3. Raman characterization of hybrid clusters without analyte

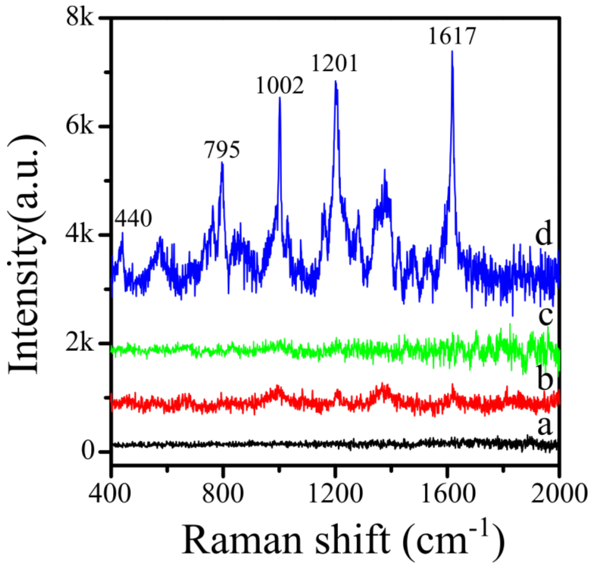

Figure S2. (a-d) SERS spectra of the magnetoplasmonic assemblies without analyte. By irradiating the sample with 0.61 mW there is no signal detected without magnetic field (a) and very weak peaks once it is applied (b). On the other hand, by increasing the power at 5.93 mW there are again no peaks visible with no magnet (c) but it is possible to distinguish clearly several features after assemblies aggregation (d).

Table S1. Assignment of the main vibrations for MG and CV.

Malachite Green	***Crystal Violet***	
cm^{-1}	*cm^{-1}*	*Assignment*
	339	ip δ(Φ - C^+ - Φ)
441	438	oop δ(Φ - C^+ - Φ)
532	527	ring skeletal vib. of radical orientation
	558	ring skeletal vib. of radical orientation
581	604	ring skeletal vib. of radical orientation
737	723	oop δ(C-H)
752	758	oop δ(C-H)
800	800	oop δ(C-H)
919	913	ring skeletal vib. of radical orientation
942	942	ring skeletal vib. of radical orientation
993	974	ring skeletal vib. of radical orientation
1175	1174	ip δ(C-H)
1222		ip δ(C-H)
1297	1298	ν(C-C)
1372	1372	ν(N-Φ)
1399		ν(N-Φ)
1444	1441	ν(C-C) + ring def.
1493	1477	ν(C-C) + ring def.
	1531	ν(C-C)
1597	1586	ν(C-C)
1621	1618	ν(C-C)

S4. Magnetic clusters

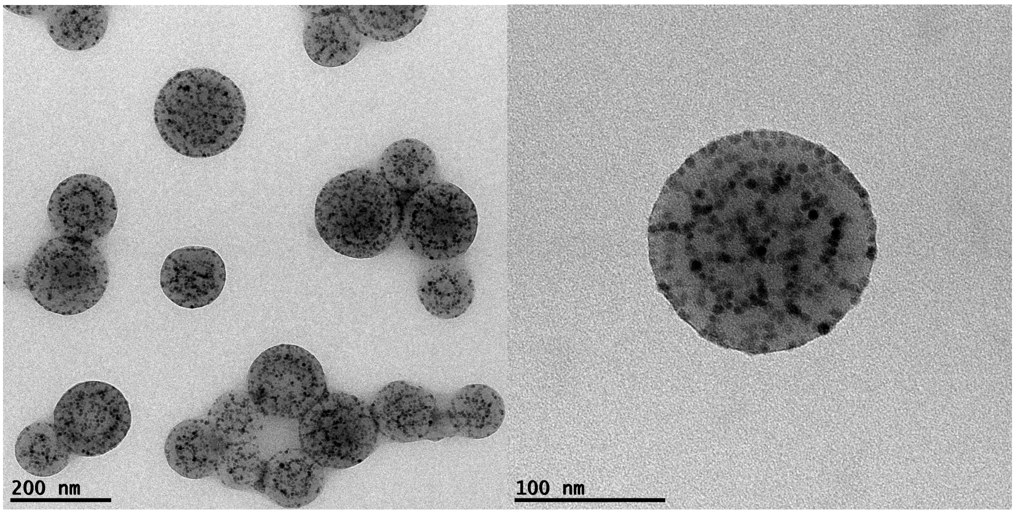

Figure S3. TEM images of magnetic Fe_3O_4 clusters without plasmonic core.

S5. Effect of analytes charge on SERS performance

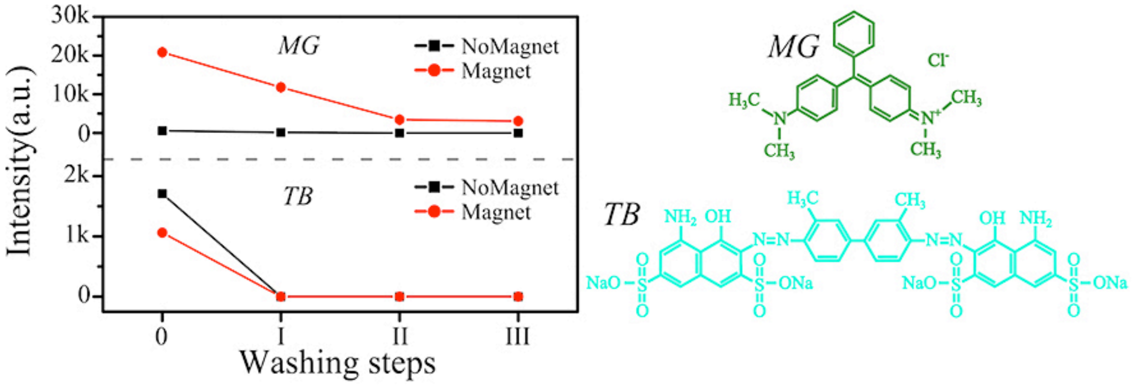

Figure S4. SERS intensity of characteristic vibrations of MG and TB (919 and 1570 cm^{-1}, respectively) vs. number of washing steps. Data were recorded before (black) and after (red) collecting the assemblies by applying an external magnetic field. Progressive decrease of the SERS intensity was observed for MG as compared to a steep drop for TB, suggesting the preferential adsorption of positively charged dye molecules on the surface of the negatively charged polymer micelles.

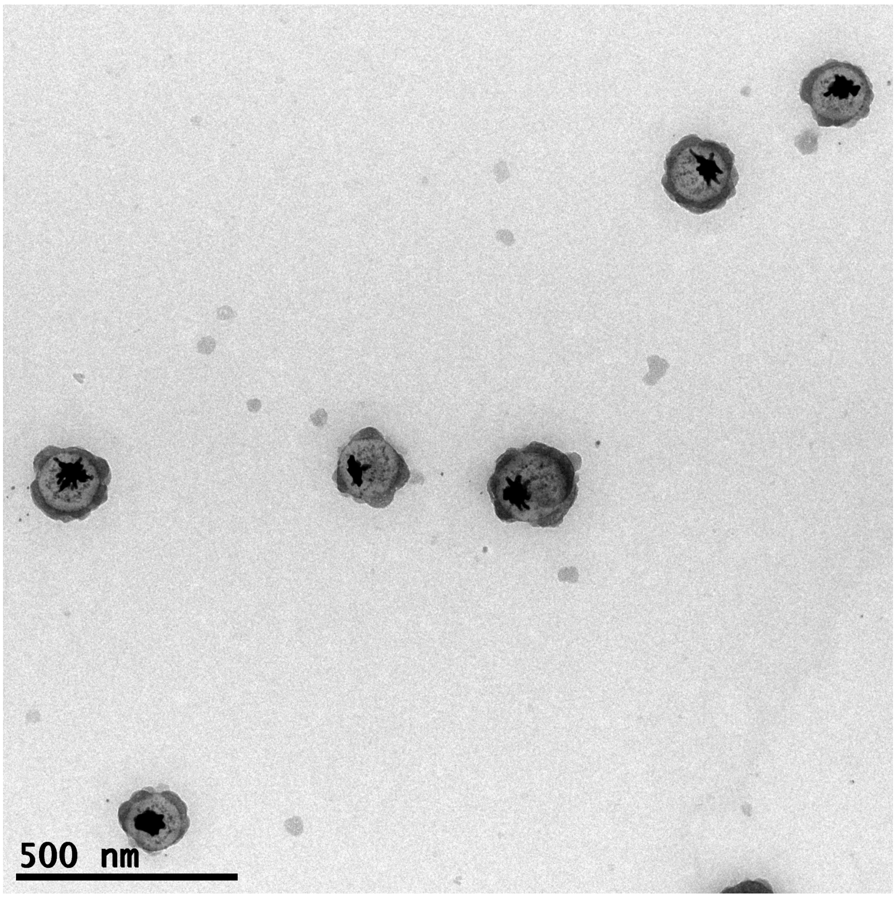

Figure S5. TEM image of the hybrid clusters after a typical SERS experiment, showing the adsorption of the positive molecular probe (MG) in the form of a molecular shell.

References

1. H. Yuan, C. G. Khoury, H. Hwang, C. M. Wilson, G. A. Grant and T. Vo-Dinh, *Nanotechnology*, 2012, **23**, 075102.
2. J. Turkevich, P. C. Stevenson and J. Hillier, Discuss. Faraday Soc., 1951, 11, 55–75.
3. J. Park, K. An, Y. Hwang, J.-G. Park, H.-J. Noh, J.-Y. Kim, J.-H. Park, N.-M. Hwang and T. Hyeon, *Nature Mater.*, 2004, **3**, 891–895.
4. W. J. Palenstijn, K. J. Batenburg and J. Sijbers, *J. Struct. Biol.*, 2011, **176**, 250-253.
5. 5. W. J. Palenstijn, K. J. Batenburg and J. Sijbers, *13th International Conference on Computational and Mathematical Methods in Science and Engineering, CMMSE* 2013, 24-27 (June, **2013**).
